# Force plate monitoring of human hemodynamics

**DOI:** 10.1186/1753-4631-2-1

**Published:** 2008-02-22

**Authors:** Jan Kříž, Petr Šeba

**Affiliations:** 1 University of Hradec Králové, Rokitanského 62, CZ – 500 03 Hradec Králové, Czech Republic; 2 Institute of Physics, Academy of Sciences of the Czech Republic Cukrovarnická 10, CZ – 162 53 Praha, Czech Republic

## Abstract

**Background:**

Noninvasive recording of movements caused by the heartbeat and the blood circulation is known as ballistocardiography. Several studies have shown the capability of a force plate to detect cardiac activity in the human body. The aim of this paper is to present a new method based on differential geometry of curves to handle multivariate time series obtained by ballistocardiographic force plate measurements.

**Results:**

We show that the recoils of the body caused by cardiac motion and blood circulation provide a noninvasive method of displaying the motions of the heart muscle and the propagation of the pulse wave along the aorta and its branches. The results are compared with the data obtained invasively during a cardiac catheterization. We show that the described noninvasive method is able to determine the moment of a particular heart movement or the time when the pulse wave reaches certain morphological structure.

**Conclusions:**

Monitoring of heart movements and pulse wave propagation may be used e.g. to estimate the aortic pulse wave velocity, which is widely accepted as an index of aortic stiffness with the application of predicting risk of heart disease in individuals. More extended analysis of the method is however needed to assess its possible clinical application.

## Background

The objective of this work is to demonstrate that force changes evoked by cardiac activity and measured by a force plate can be used to trace the motion of the heart muscle and the pulse wave propagation along the aorta. In particular the recoils caused by the pulse wave scattering on certain morphological structures (for instance on the aortal arch) can be recognized and used to determine the time points when a pulse wave reaches given location.

### Ballistocardiography

The idea itself is not new. The usage of body micro movements for extracting information about the cardiac activity is the principle of ballistocardiography – a field that has been in use for more then 50 years [1]. Nowadays body motion is usually traced by a sensitive low frequency accelerometer fastened on the sternum [2]. In the past various methods have been used, ranging from pendulous bed to induction coils. (Movements of the body occurring synchronously with the pulse were first reported by Gordon using an ordinary spring weighing machine in 1887 [3]). Similar methodology derived from the field of seismology is known as seismocardiography [4].

To elucidate the cardiac dynamics more closely a three dimensional ballistocardiography was developed during the late eighties [5]. Using a special platform the body motion was traced in three mutually orthogonal directions. More recently similar measurements have been taken also in sustained microgravity during space flights [6]. A multidimensional measurement allows a better classification of the vascular recoil, since the arteries form a three dimensional system leading to micro movements in different directions.

### Cardiac cycle and pulse wave propagation

We have studied mechanical manifestation within the cardiovascular system. Let us thus describe the pressure changes in the heart focusing on its left part. The left heart motion starts with the contraction of the left atrium which raises the left atrium pressure and leads finally to the ejection of the blood from the left atrium into the left ventricle. This process is measurable as a small pressure increase in the left ventricle. The atrium contraction is followed by the contraction of the ventricle. The ventricular pressure increases quickly and eventually exceeds the atrium pressure. This leads to a back flow that closes the mitral valve. At this stage both the mitral and aortic valves are closed. The continuing rapid ventricular contraction (isovolumic contraction) leads to an increase of the pressure that eventually opens the aortic valve and ejects the blood into the aortic root. A similar process (with a possible small time delay) takes place also in the right heart. However the pressure changes are much smaller there and result in minor effects to the ballistocardiogram.

The blood passes through the aorta into many smaller-sized arteries. The walls of the aorta and its main branches contain a high proportion of elastic materials – collagen, elastin and smooth muscles. The wall thickness of these arteries is small in comparison to their large lumen. For these reasons large arteries can be seen as thin-walled elastic tubes [7,8]. It is well-known that the liquid flow in an elastic tube is associated with the pressure (or pulse) wave propagation. At a tube ramification or its rapid bending the incoming pulse wave is scattered – partially reflected, partially divided into two or more branches. For details we refer to [9] and references therein.

During the diastolic part of the cycle the heart muscle relax and is passively filled with blood. In the left ventricle pressure changes the ventricular diastole is characterized by two main events. It starts with the closure of the aortal valve, which is recognized as the dicrotic notch in the aortal pressure signal. During the first part of diastole the ventricular pressure falls down rapidly. This pressure drop stops at the moment when the mitral valve opens and the blood flows into the ventricle – passive ventricular filling. The atrial diastole precedes the ventricular and is taking place already during the ventricular systole, so is superimposed by it.

The cardiac cycle is triggered by the electric activity represented in the ECG signal. A typical ECG tracing of a normal heart beat consists of a P wave, a QRS complex (sequence of three waves) and a T wave. Electrical systole of the atria begins with the onset of the P wave, the QRS complex is a trigger for the ventricular contraction. The T wave represents the repolarization of the ventricles.

## Methods

Standard ballistocardiography is established on registering the *motion *(or acceleration) of the body. We use another technique based on direct measurements of *forces and moments *induced by the cardiac activity. Similar methods were described in [10]. To measure the forces we used a stiff bed mounted on the top of a standard Bertec force plate, model 4060A, equipped with strain gage transducers. The force plate is capable of independent detecting the force and moment components in three orthogonal directions with a precision of 0.1 N and Nm respectively. A volunteer was lying supine on the bed, without voluntary movements. So the recorded force and moment changes were evinced mainly by the mass motion inside his body. (Due to the relaxed position, body tremor can be neglected.) Simultaneously with the force changes the ECG signal registered by a one-channel electrocardiograph was recorded. All signals were digitized with a 12 bit AD converter Tedia UDAQ-1208 with sampling rate of 1 kHz and stored to the computer hard disk. This yielded together a seven dimensional time series that was to be inspected.

The key is however not contained in the measured data but rather in the method we used to uncover the underlying processes. We understand the time series measured by the force plate as coordinate projections of a six dimensional geometric object – a (time parameterized) signal curve. To extract the information contained in the signal curve we investigate its geometric properties. In particular we focus on the geometric invariants.

Geometric invariants do not change when the curve is rotated, translated or its parametrization is changed. This is in contrast with its coordinate projections (measured signals) that change under such transformations. It is clear that if we, for instance, slightly change the position of the measured body, the intrinsic hemodynamical process will not be affected. But the individual force components measured by the force plate will change. The geometric invariants of the signal curve will, however, remain unchanged.

In a three-dimensional space geometric invariants are usually used as shape and object descriptors and apply, for instance, in computerized object recognition [11]. The idea behind this effort is simple: the invariants set the clue that remains unchanged when the object moves, rotates or the view perspective changes. Moreover they help to distinguish different object shapes, etc. These invariants usually describe the vertices and edges of the object. In fact there is an impressive amount of various invariants used in the three-dimensional space. But not all of them are useable in higher dimensions.

Here we will use invariants known as Cartan curvatures [12], that apply naturally in spaces with arbitrary dimension. Mathematically these invariants are based on the concept of local coordinate frames associated with a curve. In a given point one axis of the local frame is tangential to the curve, the second axis represents the normal, the third is the binormal, etc. Roughly speaking, when we consider a smooth *n*-dimensional curve *c*(*t*), there is for each parameter *t *an orthogonal frame (*E*_1_(*t*), ..., *E*_*n*_(*t*)) of *n*-dimensional vectors such that the *k*th derivative *c*^(*k*)^(*t*) of the curve *c*(*t*) can be expressed as a linear combination of the first *k *vectors (*E*_1_(*t*), ..., *E*_*k*_(*t*)), 1 ≤ *k *≤ *n *- 1. Thus, the first *n *- 1 vectors (*E*_1_(*t*), ..., *E*_*n*-1_(*t*)) can be obtained by the Gramm-Schmidt orthogonalization of the first *n *- 1 derivatives of *c*(*t*). The last vector is simply the unit vector perpendicular to (*E*_1_(*t*), ..., *E*_*n*-1_(*t*)) completing a right-handed frame. The family (*E*_1_(*t*), ..., *E*_*n*_(*t*)) is called the distinguished Frenet frame. The Cartan curvatures *κ*_*i*_, *i *= 1, ... *n *- 1 of the curve *c *are defined by Frenet-Serret formulae as

$$\kappa_{i}(t)=\frac{{E^{\prime }}_{i}(t)\cdot E_{i+1}(t)}{\left\|c^{\prime }(t)\right\|},$$

where ${E^{\prime }}_{i}$, resp. *c'*, stands for the derivative of *E*_*i*_, resp. *c*, and · for standard scalar product in *n*-dimensional Euclidean space. The norm in the denominator of the right-hand-size of (1) is standard *n*-dimensional Euclidean space norm.

So the curvatures characterize local changes of the coordinate system related with the curve. In our case the signal curve is 6-dimensional and is characterized by 5 curvatures. (Readers not familiar with the differential geometry can see some simple examples in the Appendix.)

Mechanical events like a heart contraction or a scattering of the pulse wave on an arterial bifurcation lead to recoil and is registered by the force plate. It might be difficult to observe this response just by inspecting the time series representing the individual force and moment components. But the events change the geometry of the total signal curve. Hence they will be visible as a changeover of its invariants. We use this strategy to trace the "footprints" of the hemodynamics in the force plate signal.

## Results

A sample of 20 healthy young adult males was tested after informed consent had been obtained. They were asked to recline supine in a relaxed position without voluntary motion. The measurement with an 8 minutes data acquisition was begun always after a calm down period of at least 5 minutes.

We used (in accordance with the literature [13]) the absolute maximum of the ECG R wave as the starting point. The response of the force plate was studied for time intervals that started 300 ms before a particular R wave and terminated 700 ms after it. This time interval usually covers the whole cardiac cycle. During this time period the heart contracts and the blood ejection takes place. The corresponding pulse wave shoots along the aorta, passing its main branchings. Finally the heart muscle relaxes in the diastolic part of the cycle.

In all measured cases we discovered several clear changes (maxima) of the curvature that came up with almost constant time delays with respect to the R wave. It is natural to assume that such are linked to events related to cardiovascular system. This expectation is strengthened by the fact that the obtained time delays are similar for all measured subjects. In addition there is a clear correlation between the observed event (change in the curvature) and the size of the stature of the measured person: taller persons embody longer time delays. This fits well with the hypothesis that relates the changes of the geometry of the signal curve to the motion of the heart muscle and to the scattering of the pulse wave on various morphological structures of the arterial system. We get naturally longer time delays between the R wave and the specific scattering event for taller persons, simply because the distance to be passed by the pulse wave is longer.

To get direct evidence that supports the above hypothesis is, however, not easy. It is rather simple to observe the motion of the heart muscle (for instance with echography) and compare the results with the signal changes of the force plate. But a noninvasive observation of the pulse wave propagation is difficult. The process is very quick and the time resolution of a standard echograph fails to resolve it [14,15]. It can be noninvasively examined only by special-purpose devices that were inaccessible for us.

To find a solution we asked for the collaboration of physicians and patients from the catheterization unit of the University Hospital in Hradec Králové. Before the examination on the catheterization unit we inspected the patient on the force plate using the method described above. Later the same patient was examined with a catheter equipped with a pressure transducer. During the investigation the blood pressure inside various locations of the aorta and the left ventricle was recorded. Since the ECG signal is recorded simultaneously with the pressure it is possible to set the time lag between the R wave and the arrival of the pulse wave at a given location.

The arrival of the pulse wave is characterized by an rapid increase in local pressure. The pulse shape is however not rectangular. We can define the time of the pulse wave arrival as the point of the maximal pressure increase i.e. as the maximum of the derivative of the measured pressure signal. A similar method was used in [16], where pressures were recorded at five regions from the ascending aorta to the iliac artery.

The knowledge about the pulse wave propagation obtained invasively during catheterization enables us to verify the hypothesis that relates the observed sudden changes of the curvature of the signal curve to the internal hemodynamical process. To do this we compared the arrival times of the pulse wave measured during the catheterization with times characterizing the localizations of the curvature peaks in the same person.

The time lags between the ECG R wave and the curvature maxima vary slightly (see Discussion), and the curvature segment corresponding to the particular heart beat can contain a curvature maxima not originated by cardiovascular system (they can be e.g. the consequence of respiration movements). To suppress the effect of movements not directly time locked to ECG signal and to obtain some average positions of curvature maxima we calculated the mean signal curve by averaging of signal segments corresponding to all particular heart beats. Then we evaluated Cartan curvatures of this mean curve. Although a more correct term for these would be "Cartan curvatures of averaged signal curve" we will refer to them henceforward as averaged curvatures.

We compared the averaged curvature peaks with the pulse arrival times on the following arterial structures:

• truncus brachiocephalicus

• top of the aortal arch

• the place where the aorta comes through diaphragm

• the point of origin of renal arteries

• the bifurcation of the abdominal aorta to the common iliac arteries

• the bifurcation of the common iliac arteries into the internal and external iliac arteries

• junction of the external iliac artery and femoral artery

**Figure 1:**
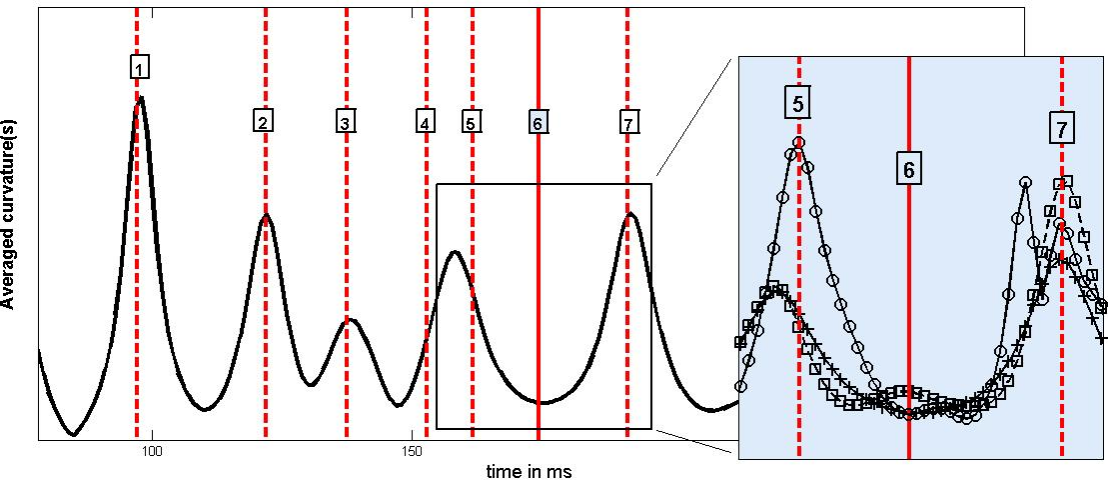
**Typical example of the first averaged curvature during the pulse wave propagation**. The first averaged curvature of the force plate signal is compared with times when the pulse wave reaches certain morphological structures (labeled vertical lines). The labels represent: 1 – aortal root; 2 – truncus brachiocephalicus; 3-top of the aortal arch; 4 – below the diaphragm; 5 – origin of renal arteries; 6 – the bifurcation of the abdominal aorta to the common iliac arteries; 7 – junction of the external iliac artery and femoral artery. The time counting starts at the maximum of the ECG R wave. The inset displays the first (crosses), second (squares) and third (circles) averaged curvatures referring to a marked time interval.

The results have been encouraging. Some of the pronounced peaks observed in the averaged curvatures appear exactly at times when the pulse wave reaches anatomically distinguished positions and is scattered. Typical results are plotted on Figure 1.

The synchronization is remarkable, especially in isolated events. When the recoil is weak it is usually not resolved by the first averaged curvature. A typical example is the arrival of the pulse wave to the aortal iliac bifurcation, where the aorta is divided into the two common iliac arteries. The pulse wave transmission on this structure is very smooth and the related recoil is small. The process is usually not visible with the help of the first averaged curvature. But it is uncovered when a second averaged curvature is used – see the inset of Figure 1. Another problematic situation occurs when there are several subsequent scatterings of the pulse wave within a very short time interval. This happens below the diaphragm. Here the pulse wave scatters on the celiac artery bifurcation, mesenteric artery and on the renal arteries. All these structures are located nearby each other. The first averaged curvature does not resolve the events and joins them into one peak, thus higher averaged curvatures have to be used. The inset of the Figure 1 shows that the third curvature resolves correctly the pulse scattering on renal arteries.

There are, however, averaged curvature peaks that appear before the QRS complex and cannot be related to the pulse wave propagation. Other averaged curvature peaks, on the contrary, refer to time delays that are longer than the time during which the pulse wave propagates along the arterial tree. We will demonstrate that these peaks are directly associated with the motion of the heart muscle.

One of the signals obtained during the catheterization is the left ventricular pressure. Since the heart motion leads to well defined pressure changes this signal can be used to trace the mechanical myocardial motion.

**Figure 2:**
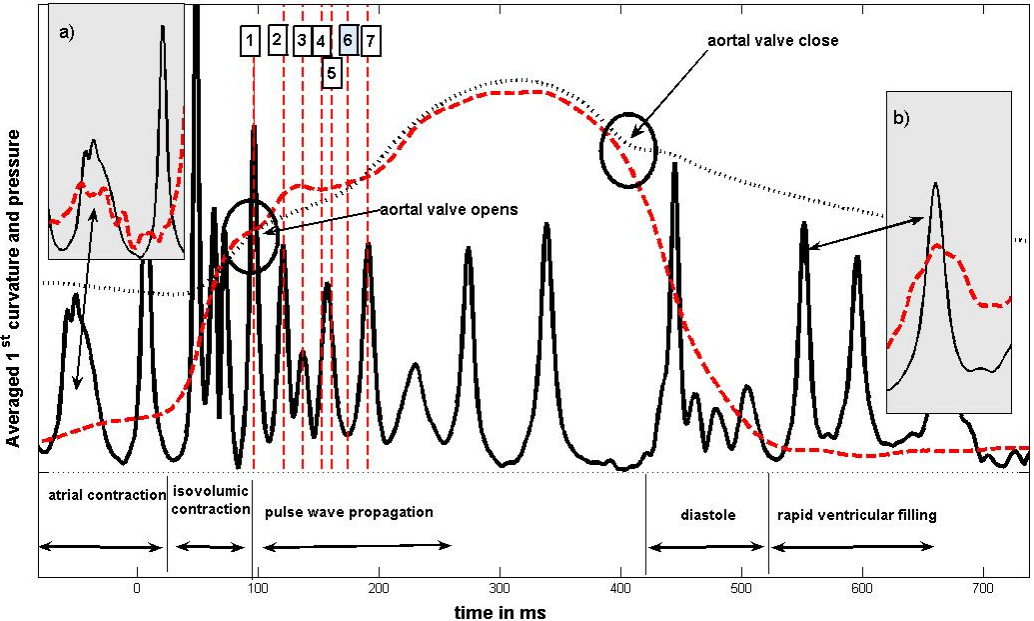
**Typical example of the first averaged curvature during the whole heart cycle**. The first averaged curvature is plotted as a function of time (full line) together with the pressure measured in the left ventricle (dashed line) and in the aortal root (dotted line). The moments when the pulse wave reaches certain morphological structures are labeled with dashed vertical lines. The numbers have the same meaning as on the Figure 1. The inset a) displays the marked peak (atrial contraction) together with the derivative of the ventricular pressure. The inset b) shows the averaged curvature peak corresponding to the rapid filling together with the derivative of the ventricular pressure.

There are usually two averaged curvature peaks preceding the ECG S wave. These peaks appear after the P wave, which marks the electric trigger for the atrial contraction. There is a clear link between the peaks and small pressure changes in the left ventricle. The first peak is related with a small increase of the left ventricular pressure caused by the atrial contraction. The position of this peak coincides with the local maximum of the derivative of the pressure signal measured in the left ventricle between the P and Q waves – see the inset *a*) in Figure 2. The second curvature peak is associated with a small plateau-like local maximum of the left ventricular pressure and follows closely the Q wave of the ECG signal. We assume that this peak reflects the force changes associated with the beginning of the ventricular systole.

The S wave is followed by a sequence of two or three averaged curvature peaks. The most important is the first of these. It marks the onset of the rapid pressure increase in the left ventricle (isovolumic contraction) and reflects the enclosure of the bicuspid valve (when a heart sound is measured this peak coincides exactly with the beginning of the first heart sound). The remaining peaks of this series are associated with heart muscle motion during the isovolumic contraction. This ends with the aortal valve opening. The valve opening is, however, not instantaneous. It starts at the moment when the ventricular pressure equals to the pressure inside the aortal root. But, as the systole continues, the ventricular pressure may – for a short time period – exceed the aortal pressure. When the aortal valve opens fully these pressures match. The opening of the aortal valve is clearly distinguished as an anacrotic notch – a bump in the measured aortal pressure. At the same time the averaged curvature of the force plate signal curve shows a sharp and high peak. It represents the aortal valve opening and the beginning of the blood ejection into the aortal root.

The referred to systolic part of the cardiac cycle is characterized by rapid heart muscle contractions and pulse wave propagation. The diastolic part of the cycle does not contain any abrupt motions. Nevertheless it is also related to an internal mass motion and hence measurable by the force plate.

The averaged curvature of the force plate signal detects the ventricular diastole as a sequence of several overlapping peaks. This sequence starts with the aortal valve closure that is marked by the dicrotic notch and/or (if the sound is recorded) by the second heart sound. It ends shortly after the termination of the sudden drop of the ventricular pressure, i.e. after the opening of the mitral valve.

This sequence of peaks is followed by one or a couple of well-pronounced averaged curvature maxima representing the first (rapid) phase of ventricular filling i.e. the phase when the blood accumulation in the atrium during the atrial diastole flows rapidly into the ventricle. This phase is followed by the atrial systole that has been already described.

An typical example of the obtained averaged curvature and pressures measured in the left ventricle and aortal root is plotted in Figure 2.

## Discussion

**Figure 3:**
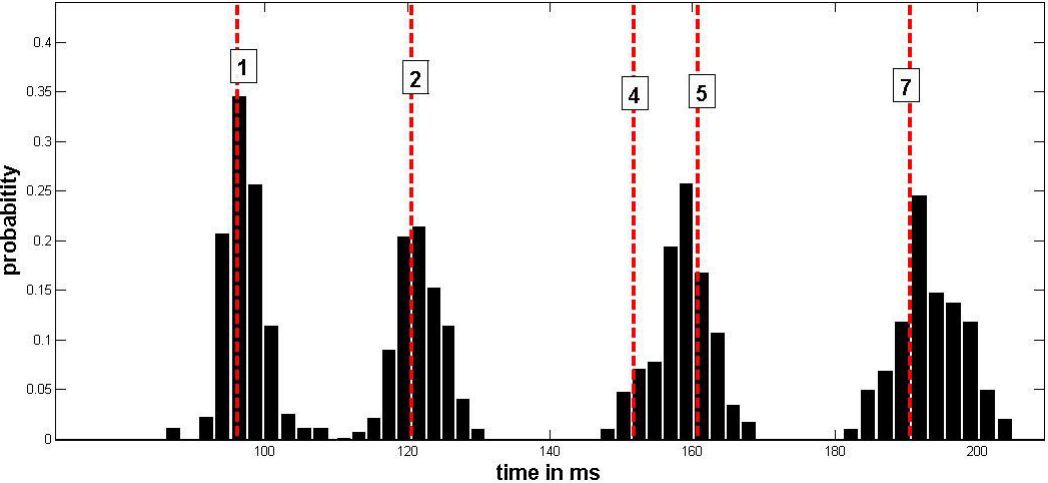
**The probability distributions of the peak localizations of the first curvature**. The probability distributions obtained during 300 subsequent heart beats of a selected individual. The distributions refer to the peaks 1, 2, 5 and 7 of the figure 1. The times of the pulse wave arrival obtained during catheterization are plotted as dashed lines.

The curvature pattern described in the previous section is reproducible. Herewith we mean the following:

• It is stable during the heart cycle. This means that the curvature peaks appear with an almost constant time delay with respect to the R wave of the ECG signal. The probability distributions of time delays referring to the peaks 1, 2, 5 and 7 of Figure 1 are plotted in Figure 3. It has been shown recently that the pulse wave velocity, which determines the positions of curvature peaks, is associated with heart rate variability [17]. To see if the width of peaks in the histogram in Figure 3 is not only the consequence of heart rate variability, we unfolded it by (linear) rescaling R-R intervals to one. The peaks became, however, broader with less pronounced spikes.

• As mentioned previously, the curvatures are geometric invariants of the signal curve. So it is not surprising that the position of the peaks is not related to the particular position of the body as long as the internal hemodynamical process is not changed. We measured the subjects in supine and prone positions. The position of the peaks is the same in both cases, but their height might vary.

• In several cases we measured the force plate response once more after a time interval of three months. The position of the averaged curvature peaks was stable, i.e. it had not changed when the same subject was measured again.

• The geometric properties of the signal curve are analogous among different people. For instance in a sample of 20 examined volunteers the curvature peak that represents the blood injection into the aortal root appeared in the mean 80 ms after the R wave with a standard deviation of 8 ms.

We used primarily the first Cartan curvature to reveal the information contained in the signal curve. Similar results were obtained also when higher curvatures are inspected. The main difference is that higher curvatures are more sensitive to the signal noise. On the other hand – as demonstrated above – some smooth processes are better visible with the help of higher curvatures.

A similar effect can be reached when the body position is changed. When the subject is measured in the prone instead the supine position some recoils are intensified and the related averaged curvature maxima are more pronounced. A typical example is the celiac artery bifurcation. It is badly visible when the subject reclines supine. But it leads to a well-pronounced peak when the measurement is done in the prone position.

## Conclusion

All this together shows that the changes of the geometric invariants of the signal curve obtained by a force plate are suitable for recording the internal hemodynamical processes. It should be stressed that the method is fully noninvasive. All that is needed is that the patient reclines quietly on a bed equipped with force transducers. Then the pulse wave propagation and the mechanical strokes of the heart muscle can be monitored in real time (online). This may be used to trace for instance the immediate influence of an administered drug, etc.

## Appendix

We discuss here for illustration the distinguished Frenet frames and Cartan curvatures in the simplest cases, i.e. in two and three-dimensional Euclidean spaces.

Let us first precisely define the notion of a curve. A (parameterized) regular curve is a smooth mapping *c *of the interval *I *into *n*-dimensional Euclidean space **R**^*n*^, such that *c'*(*t*) ≠ 0 for all *t *∈ *I*. The components of *c*(*t*) are denoted by *c*_*i*_(*t*), *i *= 1, ..., *n*.

### Plane curves

Let us find the distinguished Frenet frame and the only Cartan curvature for the case *n *= 2. Necessarily

$$E_{1}(t)=\frac{c^{\prime }(t)}{\left\|c^{\prime }(t)\right\|}=\frac{1}{\sqrt{{c^{\prime }}_{1}{\left(t\right)}^{2}+{c^{\prime }}_{2}{\left(t\right)}^{2}}}\left(\begin{array}{c} {c^{\prime }}_{1}(t)\\ {c^{\prime }}_{2}(t) \end{array}\right)$$

holds true and *E*_2_(*t*) is a unit vector perpendicular to *E*_1_(*t*) forming a right-handed frame. Thus,

$$E_{2}=\frac{1}{\sqrt{{c^{\prime }}_{1}{\left(t\right)}^{2}+{c^{\prime }}_{2}{\left(t\right)}^{2}}}\left(\begin{array}{c} -{c^{\prime }}_{2}(t)\\ {c^{\prime }}_{1}(t) \end{array}\right).$$

Vector *E*_1 _is called the unit tangent of *c*, while vector *E*_2 _the normal of *c*. The curvature is calculated using the definition (1),

$$\kappa (t)=\frac{-{c^{\prime \prime }}_{1}(t){c^{\prime }}_{2}(t)+{c^{\prime }}_{1}(t){c^{\prime \prime }}_{2}(t)}{{\left({c^{\prime }}_{1}{\left(t\right)}^{2}+{c^{\prime }}_{2}{\left(t\right)}^{2}\right)}^{\frac{3}{2}}}.$$

Note that *κ *is signed. Traveling along the curve in the direction of increasing parameter *t*, the positive value of *κ *means bending of the curve rightwards, the negative value of *κ *leftwards. It can be shown that the absolute value of the curvature equals the reciprocal of the radius of osculating circle of the curve. One can easily check both mentioned properties of the curvature on the examples of an anticlockwise, resp. clockwise, circle,

$$\begin{array}{ccc} c(t)=r\left(\begin{array}{c} \sin t\\ \cos t \end{array}\right), & \text{resp}. & c(t)=r\left(\begin{array}{c} -\sin t\\ \cos t \end{array}\right) \end{array},$$

where

$$\begin{array}{ccc} \kappa =-\frac{1}{r}, & \text{resp}. & \kappa =\frac{1}{r}. \end{array}$$

### Space curves

In the case *n *= 3 we assume in addition that vectors *c'*(*t*) and *c"*(*t*) are linearly independent for all *t *∈ *I*. One can easily find the unit tangent of *c*, *E*_1_, similarly to (2). However, the explicit forms of vecotrs *E*_2 _(principal normal) and *E*_3 _(binormal) are in general quite complicated, so we do not present them here. (Their forms become much simpler when we deal with a curve parameterized by arc-length, i.e. ||*c'*(*t*)|| = 1 for all *t *∈ *I*. It is known that any regular curve can be parameterized by arc-length.)

One can check by tedious, but straightforward calculation, that Cartan curvatures in dimension 3 satisfy

$$\begin{array}{l} \kappa_{1}(t)=\frac{\left\|c^{\prime }(t)\times c^{\prime \prime }(t)\right\|}{{\left\|c^{\prime }(t)\right\|}^{3}},\\ \kappa_{2}(t)=\frac{\left\|c^{\prime }(t)\times c^{\prime \prime }(t)\cdot {c^{\prime \prime }}^{\prime }(t)\right\|}{{\left\|c^{\prime }(t)\times c^{\prime \prime }(t)\right\|}^{2}}, \end{array}$$

where × stands for standard **R**^3 ^vector product. The first Cartan curvature is usually in **R**^3 ^denoted just by *κ *(without the subscript) and called the "curvature". It is nonnegative and equals the reciprocal of the radius of osculating circle of the curve. The second curvature is usually denoted by τ and called the "torsion". It describes the twisting of the curve, i.e. it is a measure of rotation of osculating plane (plane determined by vectors *E*_1 _and *E*_2_).

**Figure 4:**
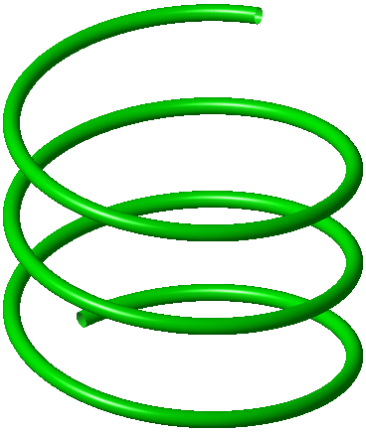
Sketch of a helix.

A nice example is a helix (see figure 4), i.e the curve

$$c(t)=\left(\begin{array}{c} \varrho \sin t\\ \varrho \cos t\\ \alpha t \end{array}\right)$$

with *ϱ *> 0 and *α *∈ **R**. By (3) we directly obtain

$$\begin{array}{cc} \kappa (t)=\frac{\varrho }{\varrho^{2}+\alpha^{2}}, & \tau (t)=\frac{\alpha }{\varrho^{2}+\alpha^{2}}. \end{array}$$

Thus, both curvature and torsion are constant. If *α *= 0 then *κ *= 1/*ϱ *and *τ *= 0 corresponding to the fact that (4) describes in such a case the circle. The sign of *α *distinguishes the right-handed and left-handed helix.

## Competing interests

The author(s) declare that they have no competing interests.

## Authors' contributions

The idea of using the differential geometry to handle multivariate time series was suggested by PŠ. Both authors worked on the improvement and final formulation of the data processing method. Both authors participated in data acquisition, data analysis and interpretation. Both authors read and approved the final manuscript.
